# Scaffold-mediated liver regeneration: A comprehensive exploration of current advances

**DOI:** 10.1177/20417314241286092

**Published:** 2024-10-13

**Authors:** Supriya Bhatt S, Jayanthi Krishna Kumar, Shurthi Laya, Goutam Thakur, Manasa Nune

**Affiliations:** 1 Manipal Institute of Regenerative Medicine, Bengaluru, India; 2 Manipal Academy of Higher Education, Manipal, Karnataka, India; 3 Department of Biomedical Engineering, Manipal Institute of Technology, Manipal Academy of Higher Education, Manipal, Karnataka, India

**Keywords:** 3D bioprinting, hepatic organoids, hydrogels, bioink, 3D scaffolds

## Abstract

The liver coordinates over 500 biochemical processes crucial for maintaining homeostasis, detoxification, and metabolism. Its specialized cells, arranged in hexagonal lobules, enable it to function as a highly efficient metabolic engine. However, diseases such as cirrhosis, fatty liver disease, and hepatitis present significant global health challenges. Traditional drug development is expensive and often ineffective at predicting human responses, driving interest in advanced in vitro liver models utilizing 3D bioprinting and microfluidics. These models strive to mimic the liver’s complex microenvironment, improving drug screening and disease research. Despite its resilience, the liver is vulnerable to chronic illnesses, injuries, and cancers, leading to millions of deaths annually. Organ shortages hinder liver transplantation, highlighting the need for alternative treatments. Tissue engineering, employing polymer-based scaffolds and 3D bioprinting, shows promise. This review examines these innovative strategies, including liver organoids and liver tissue-on-chip technologies, to address the challenges of liver diseases.

## Graphical abstract

**Graphical abstract:**
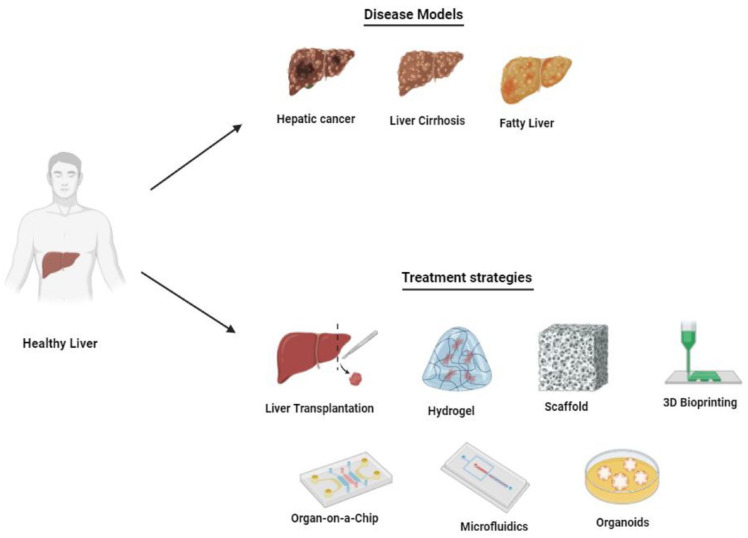

## Introduction

### Liver anatomy

**Figure 1.**
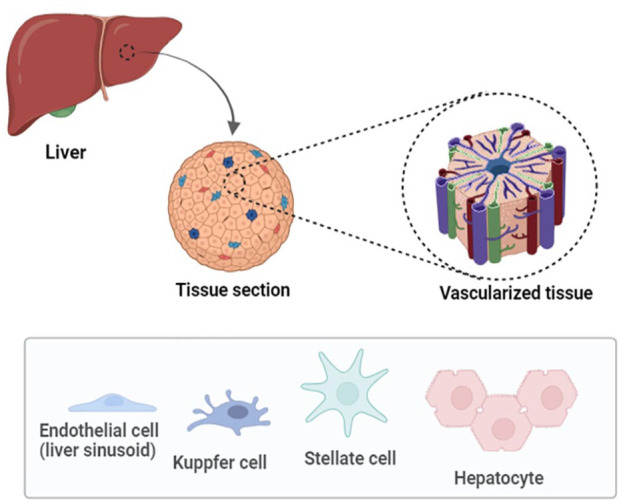
Anatomy of the liver. This figure illustrates the basic anatomy of the liver, highlighting different cell types of liver such as the Hepatocytes, Endothelial cells, Kupffer cells and stellate cells.

The Liver is the gigantic aden in the body, accounting for about 2.5% of the body’s total weight. It is situated immediately below the diaphragm at the top, on the right of the gut. The first one, the larger left, and secondly, the smaller right lobe, comprise the liver’s two main lobes. The arteria hepatica supplies oxygenated blood to the liver, while the hepatic portal vein supplies blood coming out of digestive organs that are rich in nutrients but deoxygenated. It comprises of hundreds of tiny lobules, each of which has hepatocytes-the cells that make up the liver- that are arranged hexagonally around a central vein. It is closely related to the gallbladder, which produces and stores bile, the digesting fluid generated by the liver.^1^ Hepatocytes, which comprise most of the liver’s tissue, are its principal functioning cells. They oversee many metabolic processes, including bile generation, the synthesis of proteins, and detoxification. The liver’s sinusoids are specialized capillaries that facilitate the exchange of waste products, oxygen, and nutrients between hepatocytes and blood. Specialized macrophages called Kupffer cells are found inside the sinusoids. They assist in clearing the blood of pathogens and debris. In response to injury, stellate cells, which accumulate fat in the spaces between the hepatocytes can become activated and contribute to liver fibrosis (Figure 1). The bile produced by hepatocytes is transported by the liver’s network of small bile ducts into the gallbladder for storage and, subsequently, to the small intestine to facilitate digestion.

The liver is a key component of metabolism, which includes turning nutrients from the digestive tract into stored energy. It controls blood sugar levels by releasing or storing glucose as required.^2,3^ Hepatocytes detoxify the blood by metabolizing and eliminating toxins, as well as synthesizing various proteins such as blood clotting factors (e.g. fibrinogen), albumin, enzymes, drugs, and alcohol, as well as producing bile, which is required for fat digestion and absorption in the small intestine. Kupffer cells in the liver assist in eliminating pathogens and foreign chemicals from the blood, which contributes to the body’s immunological defense.^1,4,5^

### Liver ailments

Liver diseases such as liver cancer, cirrhosis, and chronic hepatitis are major global health concerns that can have a devastating impact on people’s lives as well as the health care system and the community overall. In particular, liver cirrhosis is an end-stage illness that develops from long-term liver illnesses and is one of the causes of morbidity and demise. The prevalent disorders and diseases of the liver include cirrhosis, hepatitis, fatty liver diseases, liver cancer, and fibrosis. Liver inflammation can result from viral infections such as hepatitis A, B, C, D, and E. They can be acute (short-term) or chronic (long-term), and if untreated, they may culminate in liver cirrhosis or cancer.^6,7^ The scarring of the liver tissue that results from prolonged liver damage—often brought on by hepatitis, chronic alcohol misuse, or other illnesses—is known as cirrhosis. It affects the liver and may cause failure of a liver.^8^

When fat accumulates in liver cells, conditions including Metabolic Associated Steatotic Liver Disease (MASLD), and Non-Alcoholic Steatohepatitis (NASH) occur. They are often associated with metabolic syndrome and obesity.^9^ The most common type of primary liver cancer, Hepatocellular Carcinoma (HCC), can arise in the liver. It could start in the liver or metastasize—travel from other regions of the body to the liver. If the fundamental cause of liver scarring is not treated, hepatic fibrosis—the initial stage—can develop into cirrhosis. With the right treatment, some illnesses, like hepatitis, can be controlled or even cured, while other problems, like liver disease, may need continual medical attention or, in extreme cases, a liver transplant is necessary.

### Liver disease treatment options

**Figure 2.**
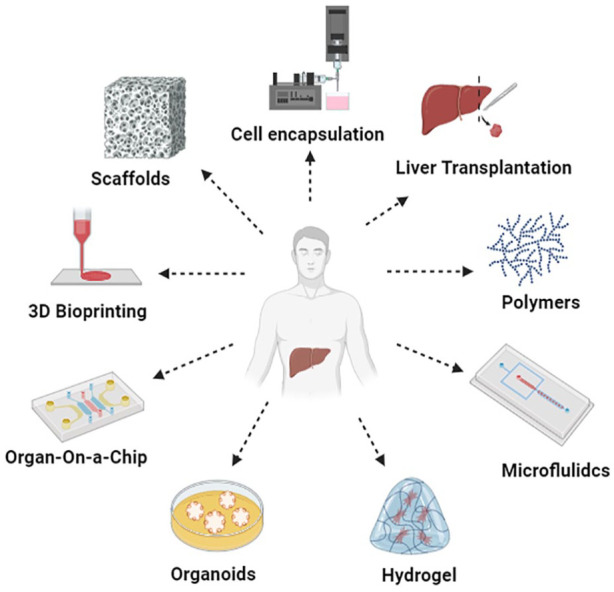
Treatment strategies for liver disorders. This figure summarizes various treatment strategies for liver disorders including liver transplantation, tissue engineering, 3D bioprinting and emerging therapies.

Liver ailments are a broad category of disorders that impact the liver, including liver cancer, cirrhosis, and hepatitis. A person’s health and quality of life may be significantly impacted by these conditions, therefore, finding appropriate treatment choices is crucial. Treatment options for liver illnesses vary based on the specific issue and degree of severity. Antiviral drugs are frequently used to treat hepatitis to suppress the virus and lessen liver inflammation. However, liver transplantation is the sole effective therapy option for the final stages of cirrhosis and liver fibrosis. The most well-established and “gold standard” medical treatment for both acute liver failure and end-stage liver disorders is liver transplantation. A healthy liver from a deceased or living donor is utilized to replace the diseased liver during a liver transplant.^10^ There are several treatment options available for certain types of liver illnesses outside of liver transplantation (Figure 2). For example, immunosuppressive medications are used to reduce inflammation and postpone the onset of liver damage in individuals with autoimmune liver diseases such as autoimmune hepatitis, primary biliary cholangitis, and primary sclerotic cholangitis.^11,12^

The primary form of treatment for alcoholic liver disease is alcohol withdrawal combined with supportive care to control symptoms and offer nourishment.^13^ Treatment for NASH and MASLD requires lifestyle modifications such as weight loss, nutrition, exercise, and taking care of underlying medical issues.^14^ In addition, specialized drugs could be recommended to treat liver disease symptoms or consequences. These therapies can manage symptoms, enhance liver function, and delay the course of the illness.^15^

### Liver disease models

Models of liver illness are essential for studying and comprehending different types of liver diseases. Because these models mimic the physiological and pathological processes associated with liver disorders, researchers can use them to investigate potential treatments, experiment with novel methods to treatment, and learn more about the underlying mechanisms behind these diseases.^10^

The cell-based model is one popular model for liver disease. In this model, the impact of medications and chemicals on liver function is investigated using cultured liver cells, such as hepatocytes. Researchers can change variables and study the cells’ responses to various stimuli in a controlled environment using these cell-based models.

Another model for liver illness is the animal model, which involves investigating liver problems in living organisms. Research on liver illness has extensively utilized animal models, notably that of rats.^16^ There are several ways to cause liver disorders in these models, including chemical exposure and genetic modification. Animal models offer a more intricate and authentic environment than cell-based models because they enable the investigation of systemic impacts, the interactions between many organs and tissues, and the impact of an organism’s physiology overall on the onset of hepatic dysfunction.^17^

The emphasis on development has increased in recent years for more advanced liver disease models that closely mimic the human liver. This includes the creation of liver-on-chip systems and extracorporeal liver support devices.^18^ These models try to mimic the liver’s complex environment, including cell-cell interactions, fluid and nutrition movement, metabolite and toxin exchange. But the use of animals in research has numerous problems, including time and expense constraints as well as ethical considerations.^16^ Therefore, the development of advanced and human-relevant liver disease models provides significant potential for advancing the knowledge of liver illnesses and creating novel therapies.^19^

### Liver tissue engineering

One promising field of regenerative medicine that aims to replace or repair damaged liver tissue is hepatic tissue engineering.^10^ Using this method, biomaterials and cells are combined to create a three-dimensional (3D) complex that closely resembles the structure and functions of the liver. Creating viable bioengineered livers for transplantation, liver disease therapy, or in vitro drug screening and disease modeling is the main goal of liver tissue engineering. The primary challenge in liver tissue engineering is the reliable and plentiful supply of functional hepatocytes, which are the main cells involved in liver detoxification, metabolism, and protein synthesis.^20^ Many cell sources have been studied in liver tissue engineering including primary hepatocytes, tumor cells, induced Pluripotent Stem Cells (iPSCs), and embryonic stem cells. But there are limitations to the usefulness, scalability, and immunogenicity of each cell source.^21^

**Figure 3.**
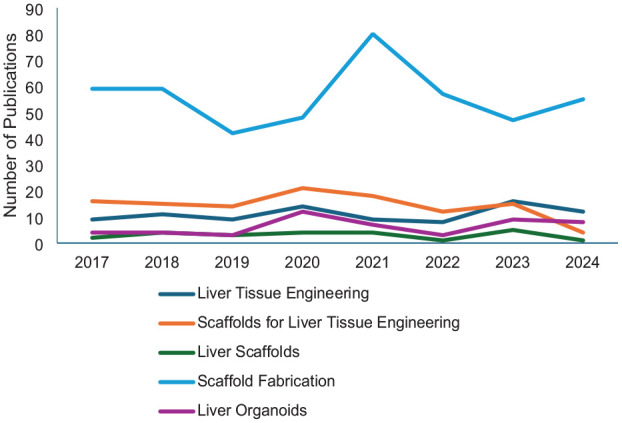
Number of publications from 2017 to 2024 derived from Scopus-advanced literature search (https://www.scopus.com) with keywords “Liver Tissue Engineering,” “Scaffolds for Liver Tissue Engineering,” “Liver Scaffolds,” “Scaffold Fabrication” AND “Liver Organoids” were used as keywords.

In recent years, there has been a notable increase in research focused on 3D-printed scaffolds and liver tissue engineering, highlighting the growing interest and advancements in these fields. To thoroughly address these developments, we conducted a comprehensive literature review on hepatic tissue engineering, with particular attention to polymer-based scaffolds, 3D bioprinting, liver organoids, spheroids, and innovative therapeutic approaches like Liver-On-A-Chip. The literature search was carried out across various academic databases and search engines, including PubMed, ScienceDirect, and Google Scholar, with a publication date filter from 2017 to 2023 to capture both foundational studies and the latest advancements. Preference was given to the most recent and relevant articles, and additional pertinent studies were identified through reference reviews (Figure 3).

## Scaffolds for liver tissue engineering

Scaffolds serve a vital purpose in liver transplantation and regenerative medicine. It creates a physical structure that enables cells to grow and organize themselves, resembling the liver’s intricate architecture.^22^ Liver on a chip system, natural and synthetic decellularized materials, and scaffolds created by 3D printing are among the scaffold types that have been investigated for liver tissue engineering.^23^

Some factors impact the choice of scaffold material, including biocompatibility, mechanical properties, degradation rate, and ability to preserve cell growth and function.^24,25^ Natural materials like collagen, gelatin, chitosan, and hyaluronic acid have excellent biocompatibility and biomimetic characteristics since their composition is comparable to the extracellular matrix of the liver. These natural materials can provide essential signals for cell adhesion and communication, facilitating cell attachment and tissue development.^20^ In liver tissue engineering, scaffold chemistry is pivotal in crafting scaffolds that serve as frameworks, offering structural integrity while supporting crucial cellular processes like attachment, proliferation, and differentiation, thus mimicking the intricate microenvironment of the liver’s natural extracellular matrix (ECM).^26^ Biocompatibility is paramount in scaffold design, necessitating accurate emulation of the native ECM to foster favorable cellular interactions.^27^ Various materials, including natural polymers such as collagen and gelatin, as well as synthetic polymers like Poly (lactic-co-glycolic acid) (PLGA) and Polycaprolactone (PCL), have been investigated for their suitability in liver tissue engineering scaffolds. Furthermore, an ideal scaffold should exhibit biodegradability, allowing gradual degradation concurrent with new tissue formation and eventual replacement by native tissue, considering the liver’s regenerative abilities.^28^ Materials such as PLGA and Polylactic acid (PLA) are often favored due to their adjustable degradation kinetics, promoting seamless integration with surrounding tissue and mitigating complications associated with non-biodegradable materials.^22^ Additionally, careful consideration of mechanical stiffness is crucial in scaffold design to achieve successful tissue regeneration, given the liver’s soft, highly vascularized nature and unique mechanical characteristics.^29^ Pore architecture and porosity are critical determinants influencing cellular behavior and overall scaffold functionality. Materials such as hydrogels, sponges, and porous ceramics are commonly utilized for their ability to support cell proliferation and tissue regeneration, closely resembling the native liver ECM.^26^ Various studies have explored optimal parameters of porosity and pore structure for liver tissue engineering scaffolds, emphasizing their crucial role in directing cellular fate. For instance, Lee et al. investigated the impact of pore size and interconnectivity on liver cell growth and function within a 3D-printed scaffold, highlighting the enhanced cell viability and metabolic activity with interconnected pores ranging from 100 to 300 μm.^30^ Similarly, Ma et al. examined scaffold porosity’s influence on the differentiation of human induced pluripotent stem cells (hiPSCs) into hepatocyte-like cells, demonstrating efficient differentiation with porosities ranging from 70% to 90%.^31^

Synthetic polymers, which include polyvinyl alcohol (PVA), PLA, polyglycolic acid (PGA), and polyethylene glycol (PEG), provide tuneable characteristics as well as controlled degradation rates. These materials can be engineered with appropriate mechanical properties to suit the intended use and provide structural support for cell growth. Decellularized materials, generated by eliminating cellular components from an organ or tissue, provide an additional advantage in liver tissue engineering.^32^ Decellularized liver scaffolds preserve the complex architecture and composition of the natural liver extracellular matrix, which is essential for the attachment of cells and proliferation also these scaffolds have been observed to boost the proliferation and differentiation of hepatic progenitor cells, making them an appealing option for liver regeneration.^33–35^

### Hydrogel-based scaffolds

Liver tissue engineering has emerged as a significant regenerative medicine field, with the potential to overcome the constraints associated with conventional liver transplantation. One of the most important aspects of hepatic tissue engineering processes is the development of appropriate support structures that enhance liver cell proliferation and activation.^21^

Hydrogel-based scaffolds are extremely intriguing owing to their enormous potential for liver tissue engineering. Hydrogels are networks of three dimensions of polymers with hydrophilic properties that can soak up a significant amount of water while maintaining structural integrity.^36^ They contain a high-water content, similar to the ECM that exists naturally in the tissue of a liver, which creates a favorable and friendly environment for cellular growth and activity.^36^ Furthermore, hydrogel-based scaffolds can be designed to mimic the physical and biochemical signals that exist in the natural liver microenvironment, which are essential for stimulating cellular behaviors such as cell adhesion, migration, differentiation, and tissue organization.^10^

There are many examples of hydrogel-based scaffolds used for liver tissue engineering. One example is the usage of gelatin-methacryloyl (GelMA) hydrogels. These hydrogels are made from gelatin, a biopolymer that is naturally present in collagen and are treated with methacryloyl groups to improve their mechanical characteristics and stability.^37^ GelMA hydrogels have been demonstrated to enhance liver cell proliferation and differentiation, as well as the development of liver-like structures in vitro. Another example is the incorporation of sodium alginate with hydrogels in liver tissue engineering.^38^ The sodium alginate compound, is a natural polymer generated from brown seaweed when amalgamated with divalent cations such as calcium, produces a gel-like consistency. Sodium alginate hydrogels have been utilized to encapsulate liver cells, offering an ideal environment for their survival and activity. In addition to these examples, PEG hydrogels have been extensively studied as common matrix materials for liver tissue engineering. PEG hydrogels have several benefits, including excellent biocompatibility and configurable mechanical properties. These hydrogels can be treated with specific ligands or growth factors to improve cell attachment, stimulate cellular interactions, and govern cell behavior in a controlled way.^39,40^ Hydrogel-based scaffolds provide various advantages for liver tissue engineering.^41,42^

### Nano-hydrogel based scaffolds

Nanostructured hydrogels have emerged as an appropriate approach to liver tissue engineering and are made up of nanoscale structures, which possess numerous benefits for liver tissue regeneration.^20^ They create an environment conducive to liver cell growth and proliferation. Nanostructures’ tiny size and high surface area-to-volume ratio enable effective nutrition and oxygen diffusion, as well as waste disposal, within hydrogels. For example, cryogels have been discovered to increase hepatocyte growth and function in vitro.^43^ Researchers have revealed that synthetic hydrogels generated by mixing decellularized extracellular matrix (dECM) and PEG diacrylate nanoparticles can help in the development of functional liver tissue constructs.^44^ Furthermore, nanostructured hydrogels can mimic the liver’s native ECM.^28^ They can be engineered to mimic the hierarchical arrangement of native liver tissue, including concentration gradients of growth factors, cytokines, and other signaling chemical substances. This biomimetic technique creates a more natural microenvironment for liver cells, by improving their ability to survive, differentiate, and effectiveness.^45^ Additionally, nanostructured hydrogels can be tailored to include bioactive compounds like growth factors or medicines for controlled release. In one instance, researchers were able to successfully incorporate hepatocyte growth factors (HGF) into nanostructured hydrogels, increasing hepatocyte proliferation and differentiation.^46^ In Another study use of self-assembling peptide hydrogels for liver tissue engineering, which contains proangiogenic moieties that promote the genesis of vascular structures in the generated tissue was explored.^47,48^ Nanostructured hydrogels have shown potential for improving liver cell proliferation, function, and vascularization. Furthermore, these nanostructured hydrogels can be created to have adjustable mechanical properties, allowing for the control of cellular behavior.^45,49^

### Decellularized extracellular matrix (dECM) based scaffolds

**Figure 4.**
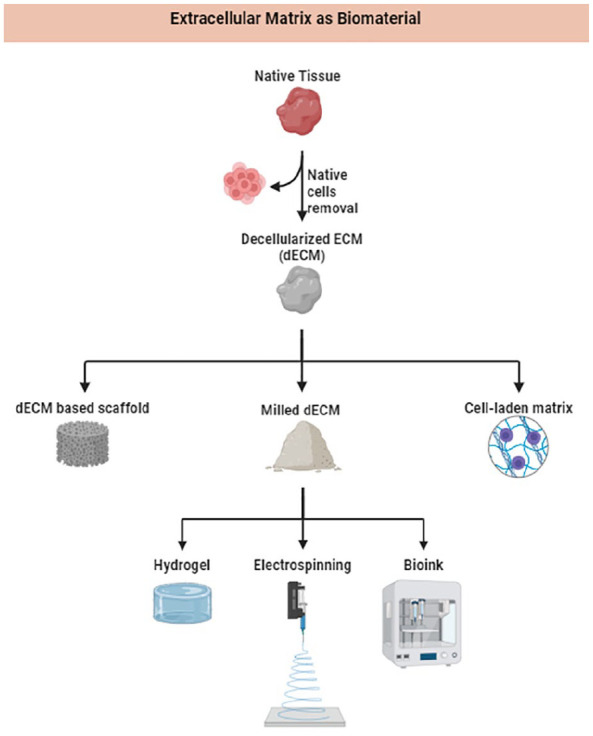
Decellularized extracellular matrix (dECM) based scaffolds. Created by Joao Vieira and Daid Ahmad Khan by biorender.com. This figure showcases different types of dECM scaffolds used in hepatic tissue engineering. dECM scaffolds retain the native extracellular matrix structure and are utilized to support cell attachment and growth. Various methods of dECM preparation and their fabrication techniques in liver tissue regeneration are illustrated.

dECM based scaffolds are formed by eliminating cellular components from tissue, retaining only the ECM framework (Figure 4).^50^ The ECM is vital for cell adhesion, polarity, proliferation, differentiation, and improving liver functions.^10^ Cryogels are an example of dECM based scaffolds used in liver tissue engineering. In one of the studies, Cryogels are created by mixing dECM with poly N-isopropyl acrylamide (pNIPAAm).^51–53^ This combination has been shown to retain hepatocyte activity in cell sheets while also promoting the adherence, expansion, and differentiation process of adipose tissue-derived mesenchymal stem cells.^54^ Another example is the application of decellularized liver scaffolds in conjunction with vascular endothelial growth factor delivery systems. These scaffolds have demonstrated positive results in boosting the creation of functional tissue constructions and improved vascularization during liver regeneration.^55^ Furthermore, researchers treated mouse livers after decellularization to create hepatic hydrogels.^20^ These hepatic hydrogels have been found to stimulate primary hepatocyte proliferation and functioning, as well as stimulate bile canaliculi development and reduction in liver fibrosis. Also, researchers investigated the utilization of decellularized liver matrix coated on 3D scaffolds.^56^ These decellularized liver matrix-coated scaffolds have good biocompatibility and have been proven to influence stem cell development while also promoting HepG2 cell proliferation.^29^ The dECM scaffolds create a natural, bioactive environment that closely resembles the original liver ECM. This promotes cell adhesion, proliferation, and differentiation, resulting in enhanced tissue development and functionality. It also has minimal immunogenicity, making it ideal for transplantation without the risk of immunological rejection. In addition, dECM scaffolds contain a 3D structure that facilitates the organization and alignment of cells, supporting tissue regeneration.^57^

## Polymer based scaffolds

Polymer scaffolds derived from biodegradable polymers have been greatly researched and developed as a technique for repairing injured liver tissue.^25^ Using polymer scaffolds, researchers can construct a framework that replicates the original ECM of the liver and facilitates the attachment, expansion, and distinction of liver cells.^43^ These scaffolds offer mechanical support to the growing tissue, preserving its structural integrity.^58^ Polymer scaffolds can be created with pore diameters and porosity, allowing for the efficient distribution of vital nutrients, oxygen, and waste materials from one cell to another within the scaffold, which is critical for the survival and function of liver cells.^59^ Also, these scaffolds can be designed with varied degradation rates, allowing for the regulated release of bioactive compounds or medicines that promote cell proliferation and liver tissue regeneration (Tables 1a, 1b). To achieve optimal cell attachment, the scaffold’s surface can be modified with ECM proteins such as fibronectin, collagen type I, and gelatin.^60–62^

**Table 1a.** Types of scaffolds. This table categorizes natural types of scaffolds used in liver tissue engineering. Scaffolds are classified based on their composition into natural polymers. Each scaffold type is described with its specific properties, advantages, and typical applications in liver tissue regeneration.

Scaffold type	Key characteristics	Raw materials	Reference
Alginate-based Scaffolds	- Biocompatible, encapsulates cells	Alginate from brown algae	Naahidi et al.^42^, Mora Boza et al.^69^, Samrot et al.^70^, Ghahremanzadeh et al.^73^, Carpentier et al.^74^, Rashid et al.^75^
	- Customizable mechanical and biological properties		
Chitosan-based Scaffolds	- Scalloped structure for cell attachment and growth - Biocompatible, biodegradable	Chitosan from exoskeleton of crustaceans	Afewerki et al.^76^, Nokoorani et al.^77^, Brovold et al.^78^, Mahnama et al.^79^, Lewis et al.^80^, Rizwan et al.^81^
Gelatin-based Scaffolds	- Similar composition to collagen, promotes cell adhesion	Gelatin (derived from bovine/porcine collagen)	Rizwan et al.^81^, Ergun et al.^82^, Lewis et al.^83^, Kim et al.^84^, Bao et al.^85^, Rupert et al.^86^, Kim et al.^87^, Ye et al.^88^, Supriya Bhatt et al.^89^
Decellularized ECM Scaffolds	- Preserves native ECM architecture and biochemical cues	Decellularized caprine/porcine liver tissue	Saviano et al.^90^, Lin et al.^91^, Mohanty et al.^92^, Sarkar et al.^93^, Gao and Callanan^94^, Ma et al.^95^

**Table 1b.** This table categorizes synthetic types of scaffolds used in liver tissue engineering. Scaffolds are classified based on their composition into synthetic polymers. Each scaffold type is described with its specific properties, advantages, and typical applications in liver tissue regeneration.

Scaffold type	Key characteristics	Raw materials	Reference
Polyvinyl alcohol (PVA) Scaffolds	- Mechanical properties resembling native liver tissue	PVA	Sasaki et al.^96^, Pilipchuk et al.^97^, Yang et al.^98^, Chen et al.^99^, Huling et al.^100^
	- High porosity and interconnected pore structure		
	- Biocompatible, low toxicity		
Polycaprolactone (PCL) Scaffolds	- Biodegradable with mechanical strength	PCL	Hosseini et al.^101^, Qutachi et al.^102^, Li et al.^103^, Yu et al.^104^, Jiao et al.^106^
	- Used for 3D capillarized tissue constructs		
Poly lactic-co-glycolic acid (PLGA) Scaffolds	- Biocompatible, promotes cell adhesion and proliferation	PLGA	Alejandro Chanes-Cuevas et al.^109^, Cauda and Canavese^110^, Zhang et al.^111^, Parlato et al.^114^, Shi L et al.^115^, Lee et al.^116^
	- Potential for vascularization in liver tissue engineering		
Polyethylene glycol (PEG) Scaffolds	- Biocompatible, tuneable mechanical properties	PEG	Ye et al.^118^, Miguez et al.^119^, Mobarra et al.^120^
	- Ease of fabrication, can incorporate bioactive molecules		

### Alginate based scaffolds

Alginate, a polymer generated from brown algae, has several characteristics that make it ideal for scaffold creation in liver tissue engineering. It is biocompatible, which means that it doesn’t trigger an adverse immune reaction or toxicity when implanted in the body. It can also enclose cells within its 3D structure, providing a protected environment for cell growth and differentiation. It is easily modified to improve its mechanical and biological properties, allowing scaffolds to be tailored to meet specific tissue engineering requirements. Several studies have demonstrated the usefulness of alginate-based scaffolds in liver tissue engineering.^38^ M. Dvir-Ginzberg et al. shown that alginate may be used as an in vitro scaffold for hepatocytes during liver tissue engineering^63^ R. Yao et al. demonstrated in vitro adipogenic differentiation by encasing human adipose-derived adult stem cells (hADSCs) into alginate & alginate/gelatin microspheres. N. Lin et al. also described in vitro differentiating of human bone marrow-derived mesenchymal tissue stem cells becoming hepatocyte-like cells utilizing the alginate scaffold.^64^ Furthermore, alginate-based frameworks have been shown to enhance hepatocyte survival and function. Chung et al.^65^ recommended improving the short-term vitality of hepatocytes cultivated on alginate/chitosan scaffolds, whereas Seo et al. observed a long-term increase in hepatocyte function in alginate/chitosan scaffolds.^66,67^

### Chitosan based scaffolds

Chitosan, a biomaterial with exceptional characteristics, was chosen as a scaffold for hepatocyte culture due to its morphology resembling glycosaminoglycans (GAG’s) found in the liver’s ECM.^68,69^ Chitosan’s scalloped shape and capacity to encourage cell adhesion and proliferation make it an excellent candidate for tissue engineering, allowing the cultivation of hepatocytes, fibroblasts, and cartilage cells.^70^ The significant properties that make it suitable for tissue engineering matrices include biocompatibility, biodegradability without toxic byproducts, reactive group availability, nontoxicity, antimicrobial properties, ease of chemical modification, high affinity to proteins, and films, fibers, porous scaffolds, and tiny spheres may all be easily fabricated.^71^ In recent years, chitosan gelatin scaffolds are being examined for hepatocyte engineering.^72^

### Gelatin based scaffolds

Gelatin-based scaffolds have gained prominence due to their high biocompatibility and biodegradability, as well as their similar composition and biological capabilities to collagen, a key component of the liver’s extracellular matrix.^73^ Gelatin, being a denatured version of collagen, exhibit strong cell attachment because of the availability of many arginyl-glycyl-aspartic acid (RGD) motifs that allow for efficient binding and contact with cells. Furthermore, gelatin-based scaffolds have been demonstrated to increase fibroblast adhesion and proliferation, making them appropriate for stimulating cell development and tissue regeneration in hepatic tissue engineering applications.^74^ Also, gelatin-based scaffolds are recognized for their rapid breakdown, which is useful in tissue regeneration. Gelatin-based scaffolds have also been combined with other materials, such as silk fibroin, to form conglomerate scaffolds for hepatic tissue engineering.^75^ These interpenetrating polymer composite scaffolds provide improved mechanical qualities and can be manufactured utilizing a variety of processes. The biocompatibility assures that they are well tolerated by cells and tissues, reducing the possibility of rejection or adverse reactions, while the biodegradability allows for gradual tissue regeneration and repair as the scaffold degrades over time.^76^ Integrin-binding regions in gelatin enhance cell adhesion and differentiation, allowing for the development of functional liver tissue. Furthermore, the wide-porous texture of gelatin-based scaffolds allows cells to penetrate the matrix bulk and adhere to the inner surfaces of pore walls, which is crucial for appropriate cell proliferation and tissue development.^77–81^

### Decellularized extracellular matrix (dECM) based scaffolds

The use of decellularized extracellular matrix-based scaffolds represents an appropriate approach in hepatic tissue engineering. Decellularization refers to the elimination of the cellular components from native liver tissue, leaving only the ECM.^82^ The decellularized liver scaffold preserves the ECM’s intricate 3D architecture and composition, including vasculature and bile ducts.^83^ It provides a natural and biocompatible environment for the implantation of hepatocellular and non-parenchymal liver cells to restore functional liver tissue.^84^ Furthermore, to maintain the integrity of the hepatic-specific extrinsic matrix, components allow for the conservation of crucial biochemical cues and signaling pathways that are essential for cell attachment and tissue formation. This method offers the potential to accurately recreate the liver’s intricate microstructures and biochemical properties, thereby improving the functionality and viability of the created tissue.^85^ Furthermore, using decellularized liver scaffolds allows for the use of allograft-derived scaffolds, which are more therapeutically relevant. Using decellularized liver scaffolds from human organs, researchers could produce a scaffold matrix that closely replicates the native liver tissue microenvironment. This can promote cell adhesion, polarity, proliferation, and differentiation while also promoting liver-specific processes including cytochrome P450 activity.^86^ Furthermore, the availability of human organs for decellularization is restricted, necessitating the utilization of organs from different species. Another possible alternative is to employ decellularized liver scaffolds from different species, such as porcine or bovine. These scaffolds can nevertheless create an appropriate milieu for the seeding of human liver and non-parenchymal cells, allowing the regeneration of functional liver tissue. Decellularized extracellular matrix-loaded scaffolds of liver tissue engineering provides various benefits.^87^

### Polyvinyl alcohol scaffolds (PVA)

PVA based scaffolds are commonly employed in liver tissue engineering due to their favorable characteristics and biocompatibility.^88^ PVA is a synthetic polymer that may be converted into porous scaffolds that give cells a 3D structure to grow and mature into functional liver tissue. These scaffolds have shown great promise for liver tissue engineering because they contain numerous important properties that are critical to the development of the engineered tissue.^89^ For starters, PVA scaffolds have mechanical qualities that are quite similar to native liver tissue. Li et al. found that PVA scaffolds had the optimal stiffness and elasticity for a healthy human liver, with an estimated range of 400–600 Pa. This guarantees that the scaffold can give the necessary support and flexibility for the cells to function properly.^90,91^ Furthermore, PVA scaffolds have a high porosity and linked pore structure, which promotes efficient nutrition absorption and waste disposal inside the designed tissue.^88^ PVA scaffolds’ interconnected pore structure enables cell infiltration and distribution across the scaffold, allowing for cell-cell interactions and the creation of functional liver tissue and these scaffolds have been demonstrated to be highly biocompatible and low in toxicity. Studies have shown that PVA scaffolds do not cause substantial inflammatory or immunological responses, making them ideal for transplantation and integration with host tissues also these scaffolds can be easily constructed into various shapes and sizes to fulfill the specific needs of liver tissue engineering.^92^

### Polycaprolactone (PCL) scaffolds

PCL is a synthetic biocompatible polymer that has received a lot of attention in liver tissue engineering because to its beneficial qualities such biodegradability, mechanical strength, and ease of production.^45,93^ This permits the scaffold to keep the deposited bioinks stable in the PCL channels, making it easier to fabricate complex and intricate tissue constructs.^94^ Ma et al. achieved the construction of a 3D capillarized tissue construct for hepatic tissue engineering utilizing PCL scaffolds.^95,96^ In this study, a combination of specialized bioinks containing three different cell types were deposited onto preprinted PCL scaffolds to simulate the vascular network of liver tissue.^97,98^ The researchers demonstrated robust cell attachment, proliferation, and differentiation within the scaffold, indicating the capacity of PCL scaffolds for producing viable vascularized liver tissue constructions.^99,100^

### Poly (lactic-co-glycolic acid) scaffolds

PLGA has emerged as viable scaffold substance for liver tissue engineering. Its strong mechanical force and excellent biocompatibility make it a good fit for this purpose.^101^ PLGA scaffolds turned out to be extensively researched for liver tissue engineering due to their favorable features. For example, PLGA scaffolds have been demonstrated to increase cell adhesion and proliferation, which aids in growth and regeneration of liver tissue. In a study, collagen coated PLGA scaffolds were created to simulate the 3D microenvironment of the original liver.^102^ These scaffolds helped mesenchymal stem cells survive and express more liver-specific genes. Another investigation by Jun Li et al. found that PLGA scaffolds paired with HGF improved liver tissue regeneration in rat mold of fulminant hepatic failure.^103,104^ The study found a rise in liver function markers such as albumin and urea production, as well as enhanced liver structure and function, when compared to control groups. Furthermore, PLGA scaffolds have been studied for their ability to promote vascularization in liver tissue engineering.^105^ Because of their hydrophobicity, scaffolds might be difficult to adhere cells to substrate. Researchers investigated various techniques to improve cell adhesion on PLGA scaffolds.^106,107^ One method is to change the surface of PLGA scaffolds by adsorbing or conjugating ECM proteins or cell-binding peptides. Wu et al. reported various attempts to improve cell adhesion on PLGA scaffolds utilizing biomaterials. They discovered that adding ECM proteins or cell-binding peptides to the PLGA scaffold improved cell adhesion and overall scaffold performance.^108,109^ PLGA scaffolds have also been employed actively for 3D printing of hepatic tissue regeneration. Yan et al. created mesoporous PLGA scaffolds for hepatocyte culturing via 3D printing. These scaffolds have a high porosity and linked pore structure, allowing for nutrition and oxygen passage while also removing waste products.^102^ The study revealed improved cell viability and liver-specific functions, including albumin synthesis and urea generation, in the 3D printed PLGA scaffolds.^110^

### Polyethylene glycol (PEG) scaffolds

PEG has emerged as a useful and successful polymer for liver tissue engineering.^111^ PEG scaffolds provide numerous advantages, including biocompatibility, customizable mechanical qualities, and the capacity to integrate bioactive compounds.^112^ These features make PEG scaffolds an optimal choice for liver tissue engineering applications. Several research have investigated the implementation of PEG scaffolds in liver tissue engineering and demonstrated their effectiveness.^105^ PEG scaffolds, for example, can provide mechanical support while also creating a 3D environment for the encapsulation of liver cells.^111,113^ Furthermore, investigations have demonstrated that the mechanical characteristics of PEG scaffolds can be controlled by altering the concentration of PEG.^108,114^ Mirmalek-Sani et al. discovered that an increase in the PEG concentration resulted in improved mechanical properties of the scaffold.^115,116^ In addition to their mechanical features, PEG scaffolds have other advantages for liver tissue engineering,^117^ for example, PEG is hydrophilic and neutral, making it friendly and resistant to protein adsorption and cell adhesion. Furthermore, PEG scaffolds can be changed to include bioactive compounds like peptides and growth factors, allowing for customized cellular microenvironments.^111^

Saeedi and colleagues investigated the potential of PEG polymers to create biomimetic scaffolds in liver tissue engineering.^112,118^ The researchers discovered that PEG acrylates, a form of PEG polymer, are commonly employed as hydrogel biomaterials in tissue engineering applications such as liver tissue engineering.^112^ PEG acrylates have been found to be efficient in developing scaffolds with customizable mechanical characteristics and the ability to integrate bioactive compounds such as growth factors. Another work conducted by Cui et al. Huang and colleagues examined and identified appropriate scaffold materials for hepatic tissue engineering.^23^ Their findings emphasized numerous benefits of PEG scaffolds, such as their biocompatibility and capacity to form a 3D habitat for liver cell encapsulation.^101,111^ Furthermore, PEG scaffolds are easier to fabricate and have better mechanical qualities than other scaffold materials, making them a popular alternative for liver tissue engineering.^43^ Mirmalek-Sani et al., for example, found that successfully encapsulating liver cells within PEG scaffolds increased cell survival and functionality. Finally, research have demonstrated that PEG scaffolds offer beneficial features for liver tissue engineering.^115^

## Processes in the fabrication of scaffolds

**Figure 5.**
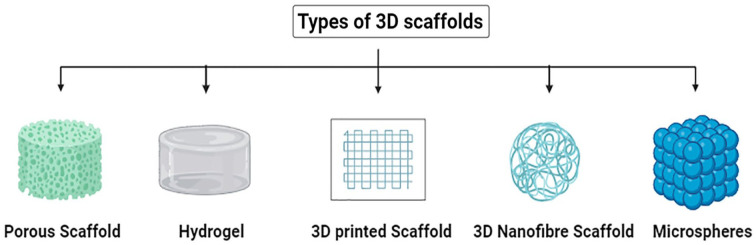
Types of 3D scaffolds. This figure presents various types of 3D scaffolds used in liver tissue engineering, including porous scaffolds, hydrogels (synthetic and natural polymers), 3D scaffolds, 3D nanofibrous scaffolds and microspheres.

Scaffolds are 3D biocompatible structures that, once implanted in the body, are intended to imitate the performance of extracellular matrices for cell adhesion and tissue regeneration (Figure 5).^121^ Pore size and porosity, mechanical and chemical qualities, polymer selection, scaffold design, and manufacturing technique are all defining aspects that affect scaffold performance (Table 2).^61^

**Table 2.** Different methods of scaffold fabrication. This table outlines the different methods used for scaffold fabrication in hepatic tissue engineering. Fabrication methods include electrospinning, freeze-drying, solvent casting, and 3D bioprinting. Each method is explained with its benefits, and limitations, as well as its suitability for creating scaffolds with specific characteristics, such as porosity and mechanical strength.

Fabrication method	Advantages	Disadvantages	Reference
Freeze-drying	- Use for a variety of purposes. - Capability of obviating high temperatures - The pore size is manageable to be controlled by changing the freezing method	- High energy consumption - The use of cytotoxic solvents - The generation of small irregular size pores	Ghahremanzadeh et al.^73^, Mao et al.^126^
Solvent casting and particle leaching	- Fits thin membranes of thin wall three-dimensional specimens - High porosity (50–90%)	- Time-consuming since thin membranes are only used - The widespread use of very toxic solvents	Grant et al.^125^, Kim et al.^127^
Electrospinning	- Essential technique for developing nanofibrous scaffolds for the TE - Homogeneous mixture of fibers with high tensile strength are obtained	- Used solvents can be toxic -Problematic to obtain 3D structures -Process depends on many variables	Samrot et al.^70^, He et al.^128^, Tappa and Jammalamadaka^129^
Fused deposition modeling (FDM)	Useful in the scaffold design under the different aspects of scaffold fabrication. Low-temperature deposition	Has limitations in its application to biodegradable polymers	Xue et al.^121^, Kang et al.^130^, Christoffersson et al.^131^
3D Bioprinting	1. Higher accuracy and greater shape complexity 2. The high speed of printing with the capability of supporting parallel high cell viability (80/90%)	It depends on existence of cells	Yang et al.43, Ye et al.^88^, Cui et al.^132^

### Conventional techniques

Conventional manufacturing techniques are responsible for establishing the paradigm for modern fabrication techniques. Despite having significant limitations, such as no control over pore size, distribution, interconnectivity, or shape, these approaches were essential for the progress of hepatic tissue engineering.^80^ These processes include Freeze-drying, Solvent casting, Gas foaming, Phase separation, and woven and spun scenarios such as Electrospinning.

Freeze drying has become a well-known approach for constructing 3D scaffolds using gels containing microspores.^65^ It was discovered that embryonic cells were more viable than 3D porous frameworks created by freeze drying, where a Gelatin-Chitosan combination was employed to explore the ramifications on HepaRG cell acetaminophen metabolism.^122^

The second technique, phase separation, is based on lyophilization, solvent casting, gas foaming, or the freeze-thaw process, which produces a different spectrum of linked pore diameters.^119^ The solvent casting procedure was employed to create pores in the 300–400 µm range for hepatocyte inoculation in a Gelatin-PLA scaffold for the control and delivery system of Fibroblast growth factor (FGF), which governs angiogenesis. Blood vessel development was detected after a week of seeding cells onto this scaffold and incubating them. This was followed by trapping in the rat’s mesentery, which enhanced the rat’s vitality by 70% following a hepatectomy.^123^

Another approach is gas foaming, which employs ammonium chloride particulates, the component of a reaction and the framework, both. This allows the scaffold to construct an open pore seeding platform, which demonstrated a greater seeding rate, boost cell survival, and albumin exudation over 2D monolayer culture and increased maturation initiation in the presence of Oncostatin M (OSM).^120^

Electrospinning is a familiar technique in generation of nano/microstructures with highly functional surfaces.^124^ The generation of Definitive endoderm from human iPSCs, which is essential for the growth of ingrained structures such as the lung, liver, and pancreas, was investigated using the electrospinning method. Furthermore, differentiation has been shown to be more significant in a 3D environment instead of 2D context, with tiny fragments such as IDE1, Activin A, and Wnt3a promoting variation.^64^ Electrospun PCL scaffolds were biofunctionalized with ECM components by stacking an ECM-producing cell layer and seeded it with HepG2 cells. The integrated scaffold beneficially impacts gene expression.^125^

### Rapid prototyping

This technology additionally referred to Layer Manufacturing or Solid free-form fabrication was created following the launch of Stereolithography (SLA) and involves layering a sample directly from a 3D design folder.^126^ This computer assisted tissue engineering approach may generate scaffolds with patient customized characteristics and microenvironment.^127^ Rapid prototyping processes are separated into two categories: subtractive manufacturing and additive manufacturing (AM). As per recent advancements, AM technology has demonstrated considerable promise with respect to constructions with elevated accuracy, high standard, high output, & little trash.^133^

**Figure 6.**
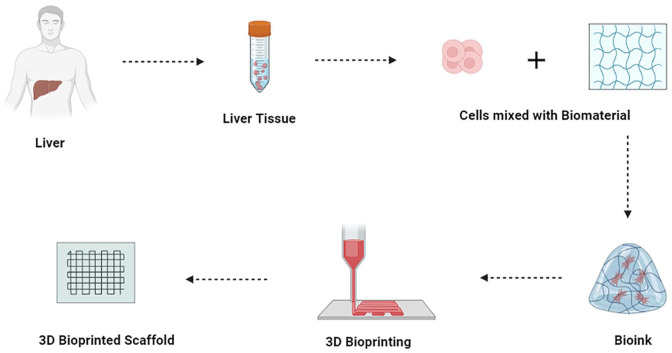
Process of 3D bioprinting. This figure outlines the step-by-step process of 3D bioprinting used in hepatic tissue engineering. The process includes bioink preparation, scaffold design, printing, and post-printing maturation.

“3D Bio-printing,” evolved from Rapid Prototyping, is an appropriate approach for generating complicated, consistent, & designed tissue and organs using a reproducible layout, such as liver. Being ability to acquire intricate arrangements may help with nutrient transfer and cellular assembly (Figure 6).^80^ Hydrogels using this technology are made using photolithography to make things with precise sizes, specific forms, and multilayer geometries, with the ability to manipulate cell position using dielectrophoretic methods.^35^

Despite being the original additive manufacturing process, SLA has remained a practical, adaptable, and accurate technique. SLA can produce scaffolds with pores as small as 20 µm, whereas other approaches can produce pores as large as 50–200 µm. The technique involves the polymerization for liquid resin in a specified sequence using spotlight or UV lamp controlled by software.^128^

A 200 µm thick lobule-like arrangement of enclosed cellular printed using digital light processing (DLP) might provide a complex microstructure and composition of cells identical to that of the hepatic. HiPSCs were encased by GelMA to create the hexagonal center hepatic cells. Human Umbilical Vein Endothelial Cells (HUVECs) and Adipose derived stem cells (ADSCs), on the other hand, had been encased in Glycidyl methacrylate hyaluronic acid (GMHA) & GelMA, which were designed with a hexagon center and periphery, respectively. When compared to hepatic 3D culture and 2D monolayer culture, this new biomimetic approach demonstrated considerable gains in function and phenotypic retention. Another study utilized DLP to create a pre-vascularized tissue having graded thickness pathways, HUVECs in the medium, and HepG2 cells around it. Endoscopic meshwork formation seen simultaneously in situ and in living tissues, including connection between the host vasculature with its transplanted vascularized tissues confirming the presence of functioning vessels.^129^

A hepatic structure was constructed utilizing 3D bioprinting technology with a 400 µm nozzle diameter and an alginate gel containing HepG2 cells. The hepato alginate framework were crosslinked to preserve its three-dimensional form and demonstrated liver-specific activity for 21 days. Another team utilized the same machine to create the same geometric form by encasing mice-derived hepatocyte-like cells in alginate (miHeps). Albumin, ASGR, and HNF4 transcription increased in vitro for 28 days, while transplanting the supporting system (scaffold) into the body improved albumin synthesis. Albumin secretion increased with time in vitro and after transplantation of the platform in vivo.^134^

FDM (Fused Deposition Modeling) seems alternative scaffold construction technology that originated in the 1990s and has subsequently been enhanced.^127^ In a study that combined PLGA with hyaluronic acid/gelatin/collagen, gelatinous formulations served to be ported to create a system originating from precipitation. Temperature, air pressure, and movement were all controlled with 100 µm dimensional accuracy in this Multiheaded Deposition System and materials were deposited on the bed via thermal and pressure in charge injectors. Polymer layers initially were set up to build scaffolds with channels for storing liquid solutions at the proper temperature. The three gels stated beforehand then went in various samples’ pathways to assess their effects, and one of it, injecting the protein collagen inside the scaffold’s tracks, resulted in strong cell adhesion.^108^ Another research investigated the impact of investing in collagen while deeply covering them onto the platform. The earlier strategy resulted in better cell activity & survival on the collagen-infused scaffold for ten days.^133^

Recent studies have resulted in major breakthroughs in Hepatic tissue engineering, which has sped up the creation of tissue constructs, recovery function, and remodeling of macro and micro liver structures to clinical standards. The evolution of conventional and 3D printing approaches in tissue engineering, especially for liver constructs, highlights their interconnected development. Initially, traditional rapid prototyping, such as SLA, utilized subtractive and additive manufacturing to create detailed, patient-specific scaffolds through liquid resin polymerization. This approach set the stage for precise pore sizes and shapes. Building upon these principles, 3D bioprinting emerged as a more advanced technique, enabling the fabrication of complex tissue constructs by layering biological materials, live cells, and hydrogels. Both methods share the fundamental principle of layer-by-layer fabrication, with 3D bioprinting refining and expanding upon SLA’s techniques to create intricate, functional tissue structures. Innovations in 3D bioprinting, including DLP and photocuring, build on the accuracy and resolution established by conventional methods. This advancement reflects a shift from simple scaffold production to sophisticated, cell-laden tissue engineering. Recent developments, such as extrusion bioprinting (EBB) and inkjet bioprinting (IBB), further enhance these capabilities, demonstrating how 3D bioprinting builds on and surpasses traditional rapid prototyping techniques to produce complex liver tissue models with functional properties.

## 3D bioprinting in liver tissue engineering

The introduction of bioprinting technology has created significant opportunities in tissue engineering. It includes the production of 3D biological constructs by layering diverse natural substances, biological molecules, and live cells, allowing for precise positioning of the components.^135^ It additionally offers multiple benefits, including the ability to produce elaborate 3D models, concurrent cellular imaging, excellent recurrence accuracy, manageability, & productivity.^51^ This chapter discusses some of the qualities and elements that impact 3D bioprinting in liver tissue engineering, such as bioinks, cell lines, and applications.

Because of the wide range of mechanisms accessible in the Additive Manufacturing industry, and the numerous combinations of bioinks and cell lines, new 3D bioprinting techniques are constantly evolving. Some of the few 3D bioprinting techniques utilized in liver tissue engineering include IBB, photocuring bioprinting (PBB), EBB and scaffold-free bioprinting (Table 3).

**Table 3.** Types of 3D bioprinting. This table provides an overview of the various types of 3D bioprinting techniques used in liver tissue engineering. The techniques include extrusion-based bioprinting, inkjet bioprinting, and photocuring based bioprinting. For each type, the table describes the principle, advantages typical applications in creating complex liver tissue constructs.

Bioprinting technique	Description	Advantages	Example applications	Reference
Extrusion-based Bioprinting (EBB)	Uses pneumatic or mechanical systems to dispense bioink layer by layer, with subsequent crosslinking to create 3D tissue structures.	- Simplicity and versatility in printing various materials	Bioprinted HepaRG-laden cellular construct using alginate bioink	Roy et al.^135^, Murata et al.^143^, Moldovan et al.^144^
Inkjet-based Bioprinting (IBB)	Adapts inkjet desktop printer technology to drop picolitre volumes of cell-laden bioink continuously or on-demand.	- High resolution (100 µm)	3D liver tissue structures combining HepG2 and HUVEC cells	Kizawa et al.^145^, Cui et al.^146^
Photocuring-based Bioprinting (PBB)	Utilizes a light source to cure light-sensitive bioink layer by layer, allowing precise control over porosity and mechanical properties.	- High precision (25-100 µm)	Multilayer 3D hepatic tissue constructions using SLA and PEGDA bioink	Nicolas et al.^147^, Lee et al.^141^, Shri et al.^148^, Tong et al.^149^

An EBB procedure was member among the earliest methods tested for the biological printing of 3D tissues architectures. To accurately regulate the overall distribution & movement within the bioink, a pneumatic or mechanical fluid dispensing system (piston or screw-driven) is utilizing conjunction alongside an automated technology.^132^ The dispensed bioink is crosslinked using light, heat, or chemical processes, allowing for the exact layering of cells enclosed within diverse hydrogels as well the creation of custom 3D tissue structures. EBB can be extruded directly or indirectly. The bioinks are immediately deposited in the direct extrusion process, resulting in 3D structures. Conversely, incident perspective prints with oblatory inks that can be removed later to reveal empty structures. Principal benefits of EBB are its clearness, adaptability in bioprinting different materials, potential in imprinting excessive cell densities, & similarity with a wide range of materials.^136^ A study used alginate bioink to create a printed HepaRG-containing cellular construct.^137^ 7 days after maturation, the cells expanded an imprinted structures developed a spherical form and acquired liver capabilities such as the breakdown of drugs, albumin production, and the storage of glycogen (in addition to the presence of 0.5% dimethyl sulfoxide).

IBB is modeled after traditional two-dimensional inkjet printers. The IBB method is a well-studied printing technology that uses precise droplets of a cell-laden bioink to create 3D tissue constructions on underlayer.^138^ Although inkjet printers extrude bioink droplets utilizing thermal, microvalve, acoustic, or piezoelectric processes, heat inkjet technology is the most often utilize for biological printing due to its simplicity, cost-effectiveness, efficiency, and excellent cell viability.^139^

IBB’s primary advantages are its excellent spatial resolution (100 µm) & capability to produce attention slants of biological materials, cells, and growth factors. A study used this technology to induce 440 µm 3D infrastructures on chip by integrating a distinctive order cell deceive appeal with independent inkjet printing. A subcaste of mortal HepG2 cells was squeezed connecting two covering of mortal HUVEC in the 3D liver model.^140^

PBB use an origin of illumination to cure a light-phobic bioink, which is latterly used to form 3D structures subcaste by subcaste. The light source that determines the design to be produced could be a ray (as in stereolithography) or a projector (as in digital light processing) This perspective permits the production of cell-loaded 3D-designed tissue constructs with mini-form infrastructures ranging from 25 to 100 µm accurate command over sponginess & mechanical properties, and no constraints on bioink density. Exceptionally, a resolution of 5 µm is also attainable using the advanced SLA bioprinting process, unlike ordinary SLA and DLP, incorporates a spatial light modulator (SLM).^141^

A study used SLA to generate layered 3D hepatic tissue structures with hepatocytes laden Polyethylene glycol diacrylate (PEGDA) bioink, it cultivated them in a perfusion bioreactor.^130^ Under perfusion conditions, the tissue constructions revealed remarkably enhanced cell use (albumin and urea secretion). An additional investigation employed SLA to print hydrogel scaffolds and install them in an infused fermenter casing, which extended the deprivation of albumin production by refined rat hepatocytes for 7 days. Experimenters discovered that the infusion rate, cell compactness, and culture surroundings (existence or nonexistence of epidermal growth factor (EGF)) all influence hepatocyte functionality in the establishment of culture.^131^ A free software tissue engineering stereolithography device (SLATE) was lately working to produce pre-invasive hepatic hydrogel porter.^142^

Despite significant advances in bioprinting, the traditional outlook of pressing cells/spheroids into a hydrogel-based bioink has certain foundational restrictions. External materials and crosslinking strategies (such as chemicals or UV) utilized in these approaches may influence cell survival. Following implantation, biomaterials may elicit inflammatory responses.^135,143^ Despite substantial advancements in bioprinting, the classic method of pressing cells/spheroids into a hydrogel-based bioink has a few foundational restrictions. Using outward substances and interconnecting techniques (i.e. chemicals or UV) in this activity perhaps reduce cell survival. Following implantation, the biological materials may exhibit inflammatory response.^144,145^

### Bioinks for liver tissue engineering

The usage of bioink formulation in bioprinting is crucial for producing hepatic tissue by utilizing this technology. These bioink compositions contain biological materials, liver cells, and additional aiding factors.^150^ Regardless of the method utilized, they must have some critical traits for effective printing, such as printability, form structural integrity, biologically compatible, and biologically active properties.^54^

Bioink compositions’ printability varies depending on the type of bioprinter employed. After printing for a defined culture period, bioink formulations must also display shape fidelity/structural stability. Bioink forms must also be biocompatible and capable of retaining in vivo-like hepatic cell activity and morphologies in printed structures. Notably, scaffold-free bioprinting techniques use cells directly as bioinks (homogeneous or heterogeneous), with no involvement of biomaterials (Table 4).^151^

**Table 4.** Bioink composition. This table lists the different compositions of bioinks used in 3D bioprinting for liver tissue engineering. Bioinks are categorized based on their base materials, the table also details the key properties of each bioink, such as viscosity, biocompatibility, and biodegradability, and their relevance in supporting cell viability and tissue formation.

Bioink composition	Critical traits for effective printing	Notable features	Example applications	Reference
Biomaterial-based	- Printability - Form fidelity/structural stability - Biocompatibility - Bioactive properties	- Utilized in various bioprinting methods - Enables customization of mechanical and biological properties	Scaffold-based bioprinting of hepatic tissue structures	Pai et al.^155^, Dzobo et al.^57^
Scaffold-free (Cell-based)	- Printability - Form fidelity/structural stability - Biocompatibility - Retention of in vivo-like hepatic cell activity and morphologies	- Involves direct use of cells as bioinks - Homogeneous or heterogeneous cell bioinks	Directional growth of bone marrow derived MSCs toward hepatic lineage in pig liver tissue bioinks	Panwar et al.^156^
Gelatin-based with ECM microparticles	- Enhanced mechanical properties Improved printability potential - Promotion of functional characteristics of cultured hepatocytes	- Incorporation of 13.4 μm ECM microparticles - 9.17 times increase in mechanical properties	Enhancement of functional characteristics in cultured hepatocytes	Hu et al.^157^

They identified directed proliferation of mesenchymal stem cells obtained from the bone marrow toward the hepato lineages using bioinks composed of pig liver tissue, instead of cells from the cardiovascular system, the cornea, or epidermis.^152,153^ Furthermore, mixing 13.4 µm pig liver ECM microparticles with a gelatin-based bioink improved the operational properties of produced hepatic cells. Furthermore, the mechanical properties & printing capability of this microparticle incorporated gelatin bioink formulation were enhanced.^154^

### Cell sources and cell-lines in liver tissue engineering

The liver is mostly made up of parenchymal and non-parenchymal cells. Non-parenchymal cells link with and assist the hepatic parenchymal cells, which perform most of its functions. The most common type of hepatic parenchymal cell is the hepatocyte.^146^ The liver contains three types of non-parenchymal cells: Kupffer cells, Endothelial cells, and hepatic stellate cells (Table 5).^147^

**Table 5.** Cell types used in liver tissue engineering. This table presents the various cell types used in liver tissue engineering, including primary cells and cell lines. The table describes the role of each cell type in liver tissue formation, their sources, and their specific functions in liver regeneration and its applications in bioprinting.

Cell type	Role	Abundance in liver	Characteristics	Applications in bioprinting	Reference
Hepatocytes	Major parenchymal cells	60% of total cells; 80% of total volume	Key liver functions: bile synthesis, glucose metabolism, drug metabolism	Best cell source for bioprinting in vitro liver tissues; Challenges with scarcity and phenotypic loss	Parak et al.^165^
Hepatoma-Derived Cell Lines (e.g. HepG2, HUH7)	Alternative to primary hepatocytes	Varied	Represent major hepatocyte functions; Used for liver microenvironment bioprinting	Extensive use for liver microenvironment bioprinting; Widely available	Lewis et al.^80^, Grix et al.^162^
Hepatic Stellate Cells	15% of total liver cells; Found in Disse’s area	Regulatory role in liver fibrosis; Activated by microenvironment changes	Co-cultured to mimic hepatic microenvironment; Aids in maintaining parenchymal cell phenotypic and functional characteristics	Contribution to liver fibrosis studies; Co-culture in hepatic microenvironment bioprinting	Krause et al.^161^
Hepatic Sinusoidal Endothelial Cells	15% of total liver cells; Located in Disse’s area	Regulatory role in liver fibrosis; Respond to microenvironment changes	Co-cultured to mimic hepatic environment; Helps maintain phenotypic and functional characteristics of parenchymal cells	Important in hepatic microenvironment studies; Co-culture applications in bioprinting	Krause et al.^161^
Kupffer Cells	Liver sinusoidal macrophages	Present in sinusoids	Antigen processing, immune response regulation, particle/toxin removal	Added to in vitro liver models for regulatory response studies in bio-printed liver tissue	

Hepatocytes: It comprises nearly all liver parenchymal cells, making up to 60% of all cells and 80% of the entire volume.^157^ They oversee critical liver activities including bile production, glucose utilization, and hazardous drug metabolism. Because of their high metabolic activity, basic liver cells, or the cells isolated straight from hepatocytes, remain appealing cellular resources in biological printing for in vitro hepato tissues.^158^ Yet, because of scarcity in human derived elementary hepatocytes, primary hepatocyte synthesis remains challenging, as the cell types are prone for losing its phenotypic features.^159^ Instead, which could represent a wide range of important hepatocyte functions such as albumin production, carbamide manufacturing, & Cytochrome 450 (CYP450) associated reaction, were commonly employed in hepatic microenvironment bioprinting. Different bio-ink components have been created to mimic in vivo microenvironment as closely as possible by spotting cell shape along with analysis that occur in the basic hepatocytes utilized for the 3D-bioprinted in vitro approach, and the physiological modifications of cells in vitro are also accurate in regard to other hepatic cell lines.^160^

Liver Stellate cells: It account for 15% from all hepatocytes and are concentrated in the Disse’s region. Under normal conditions, hepatic stellate cells rest but when their environment is changed, hepatic stellate cells became triggered, causing collagen and other related proteins to rapidly rise and play a key part in liver fibrosis. Viruses, alcohol, and an overabundance of iron can all help in this process.^161^ In other investigations, hepatic stellate cells had been co-cultivated with parenchymal cells to mimic the liver’s milieu, which helped parenchymal cells maintain their phenotypic and functional integrity.^159,162^

Endothelial Cells: Filtration is performed by the sinusoidal endothelial cells that line the walls of the hepatic sinusoid. They absorb tiny particles and contribute to pathogen elimination. They also function as antigen-presenting cells and release cytokines and eicosanoids.

Kupffer cells: This cell types tend to be macrophages found within sinuses located in the liver. Their fundamental duties include processing and transporting antigens, regulating the organism’s immune response, and removing particles and poisons from the portal vein. Kupffer cells were recently introduced into artificial hepatic framework aimed at investigating its regulatory responses with regards to bruise response in bioprinted liver tissue after cytokine and pharmaceutical activation.^163,164^

### 3D bioprinted liver tissue models

For various research purposes, numerous laboratory hepatic tissue simulations have been generated by implementing 3D biological printing to achieve hepatocellular functions.

#### Models of hepatic disease & drug screening

For simulation of Hepatic disease, scaffold-based hepatic tissue simulations can be used. Hepatitis-B Virus (HBV), Hepatitis-C Virus (HCV), cirrhosis, and hepatoma constitute one of the most common reasons for mortality worldwide, posing a significant danger for the safety of people. The use of 3D-bioprinted hepatic constructs in conjunction with primary hepatocytes could lead to an increased in precise estimation for liver damage produced by drug. All hepatic disease conditions simulations are frequently used for pharmaceutical business for reduction in drug development error rates. The drug-induced hepatic injury, hepatoma, and hepato fibrosis designs for demonstrating working of three-dimensional biological printing can be utilized for generating hepatic disease simulations^141,166^

Medicine-convinced liver toxin is leading source of medicine development failure known as the drug induced liver injury (DILI). 3D-biologically printed hepatic tissue simulations is an effective technique for assessing the hepatotoxicity of medicine during medicine webbing.^167^ 3D-bioprinted liver tissue constructions were tested with hepatocytes derived from patients & non-parenchymal cells to study the organ-position response to clinical medicine-convinced hepatotoxicity cure responses of the hepatotoxic medicine Trovafloxacin & the innocuous drug Levofloxacin.^168^ The data show that trovafloxacin can beget considerable quantity-dependent DILI at a clinically applicable cure (4 × 10^6^ M or 4 µM). This 3D-bioprinted model demonstrated a clinically meaningful response to hepatotoxic specifics. 3D-bioprinted hepatoma simulations, almost all are generally utilized liver diseased models for studying the molecular mechanisms behind the beginning of hepatoma, development, metastasis, & anticancer medicine webbing. Because of their low cost, HepG2 cells are almost frequently employed hepatoma cell source.^140,169^

DILI occurs when medications, including prescription drugs, over-the-counter drugs, and dietary supplements, cause liver damage. This condition is categorized into intrinsic and idiosyncratic types, with acetaminophen (APAP) being a notable cause of intrinsic DILI, responsible for about 50% of acute liver failure cases in the U.S. and Europe. Diagnosing DILI requires careful documentation of medication exposure, exclusion of other liver disorders through medical history, and liver biochemical tests. Although most patients recover fully, some may develop acute liver failure or chronic liver injury. Treatments focus on discontinuing the offending drug and providing supportive care, with liver transplantation considered in severe cases of liver failure. Causality assessment tools, such as the Naranjo Adverse Drug Reactions Probability Scale and the Council for International Organizations of Medical Sciences/Roussel Uclaf Causality Assessment Method (CIOMS/RUCAM) scale, help evaluate the likelihood that a drug caused the liver injury, but these tools have limitations and cannot replace clinical judgment. As understanding of DILI mechanisms continues to advance, the need for better methodologies in evaluating and managing DILI remains crucial, particularly considering the growing use of herbal and dietary supplements linked to liver injury. APAP overdose is a key model for studying DILI due to its well-characterized mechanism involving the toxic metabolite N-acetyl-p-benzoquinone mine (NAPQI), which causes liver damage through mitochondrial dysfunction. Despite advances, such as insights into cellular signaling pathways and the role of extracellular vesicles our understanding remains incomplete, highlighting the need for improved human-based models. These models are crucial for predicting DILI and developing therapies, as current animal and in vitro systems often fall short due to interspecies variability and metabolic differences. Recent developments include advanced 3D bioprinting and organ-on-chip technologies, which integrate multiple cell types and mimic liver complexity more accurately. These platforms, including humanized chimeric mice with human liver and cytochrome functions, offer a better representation of human drug metabolism and interactions. For instance, chimeric mice have been used to study drugs like fenclozic acid, though challenges such as residual murine hepatocyte activity remain. Additionally, zebrafish models provide a high-throughput, cost-effective means to study DILI due to their genetic similarity to humans and ease of imaging. Treatment strategies are evolving, with focus on utilizing novel technologies and human-based models to enhance drug safety testing. These include toxicogenomic, which integrates genomic, proteomic, and metabolomic data to improve mechanistic understanding and predict DILI. Cellular models, such as the HepaRG cell line and iPSC-derived hepatocytes, are being used to study drug-specific toxicity and simulate human liver responses more accurately. Emerging strategies also involve non-invasive imaging and multi-organ models to better capture liver pathophysiology and drug interactions. Overall, the shift toward advanced, human-relevant models and bio convergence approaches promises to improve DILI prediction, identify new therapeutic targets, and enhance personalized medicine, ultimately addressing the complex challenges of drug-induced liver damage.

Clinical monitoring for signals of liver injury, such as elevated serum alanine aminotransferase (ALT) levels, is important, as increased ALT abnormalities in clinical trials have been linked to numerous recent cases of idiosyncratic hepatotoxicity. However, compliance with monitoring is poor, and even when elevated ALT is detected, progression to acute liver failure can occur rapidly, before intervention. The experience with drugs like troglitazone and ximelagatran illustrates that signals of liver injury in clinical trials do not always accurately predict the risk of severe, life-threatening toxicity that can occur post-marketing. While animal models fail to reliably predict idiosyncratic hepatotoxicity, mechanistic insights can be gained from studying APAP hepatotoxicity. This model demonstrates the key function of the innate immune system in controlling liver damage development and severity after the initial drug metabolism. Exploring the genetic and environmental influences on the innate immune system and liver regenerative processes may provide important clues about the pathogenesis of idiosyncratic drug-induced liver injury in humans.

#### Scaffold-free spheroids

In addition to cells embedded in biomaterial scaffolds, cellular spheroids are used in a hydrogel-free 3D culture paradigm.^140^ Spheroids are self-organizing, spherical cellular aggregates that do not require scaffolding, mitigating some disadvantages of two-dimensional culture. Primary Hepatocytes (PHCs) can maintain their phenotypic and function by making spheroids, which suggests a way for producing hepatic buds without the use of biomaterials such as collagen, gelatin, and Matrigel.^140,170,171^ To produce liver bud-like spheroids, 3D bioprinting was combined with needle array technology. Hundreds of these liver bud-like spheroids were 3D printed and implanted in nude rats to investigate liver regeneration.^141,170^ The research shows that transplantable liver organoids can be created in vitro. Traditional methods for producing hepatocyte spheroids include hanging drops, microwell arrays, and magnetic assembly.^148,149,172^ Every strategy has advantages and disadvantages. Furthermore, the appropriate cell types for each technique vary due to phenotypic and in vitro cell culture properties such as spheroid diameter and stability, both of which influence the effectiveness of cell aggregation formation. Hepatocyte spheroids are scaffold-free, cell-laden hydrogel structures and hepatocytes were usually encapsulated within the hydrogel.^173^

#### 3D-Bioprinted liver-on-chips

Photolithography and replica molding are both costly and time-consuming methods for creating microfluidic liver on chips. Microfluidic liver-on-chips, 3D bioprinting can be utilized to manufacture complex cell-laden scaffolds on a chip for perfusion culture and microfluidic liver-on-chips are created quickly and cheaply.^174^ 3D heterogeneous cell-encapsulated hydrogel-based matrix can also be printed using 3D bioprinting and microfluidic biochip technology to find, develop, and test novel hepatotoxicity medications.^173,175^ 3D bioprinting allows for better control of the cell-hydrogel mixture in the biochip, which can also be molded into a more suitable shape for future research.^155^

**Figure 7.**
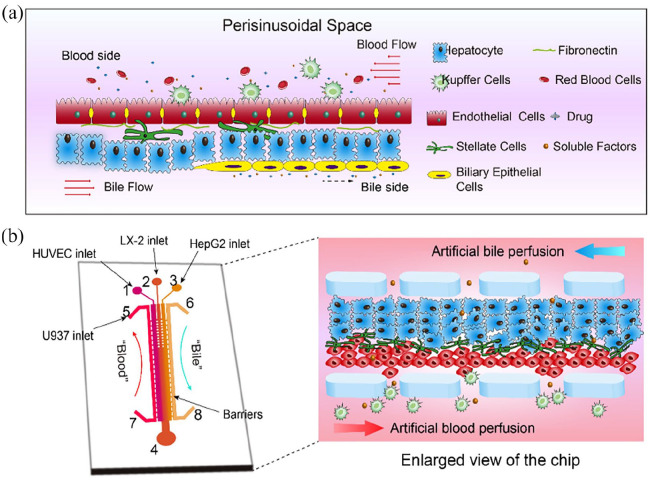
(a) micro physiological structure of Liver tissue. (b) Schematic diagram of Liver-On-A-Chip. (Reproduced from^176^ Deng et al. 2020, Biomicrofluidics 2020,^14^ under creative common attribution license CC-BY).

In Figure 7(a), a specific spatial arrangement of hepatocytes (HCs), hepatic stellate cells (HSCs), liver sinusoidal endothelial cells (LSECs), and Kupffer cells (KCs) forms the structure of the liver sinusoid. HCs are the primary functional cells that create linked liver plates. Layers of the vessel wall are made up of LSECs that are logically stacked in a row. HSCs are located in the perisinusoidal gap that separates two cell columns. KCs are found in the lumen of the hepatic blood arteries, just above the layer of LSECs. These four cell types are usually found in parallel bands in the liver, which helps with waste metabolite clearance and continuous transport of nutrients and oxygen from the portal to the central vein. Figure 7(b) simulates the hepatic sinusoid to illustrate the liver chip. A sinusoidlike structure is formed by the successive deposition of HCs, HSCs, and LSECs within the central microchannel when laminar flow regulation is in place. KCs stick to the LSECs layer’s surface. Microchannels for artificial blood and bile perfusion are built adjacent to the central microchannel, with the direction of artificial blood flow being opposite to that of bile flow.^176^

## Organoids in liver treatment

**Figure 8.**
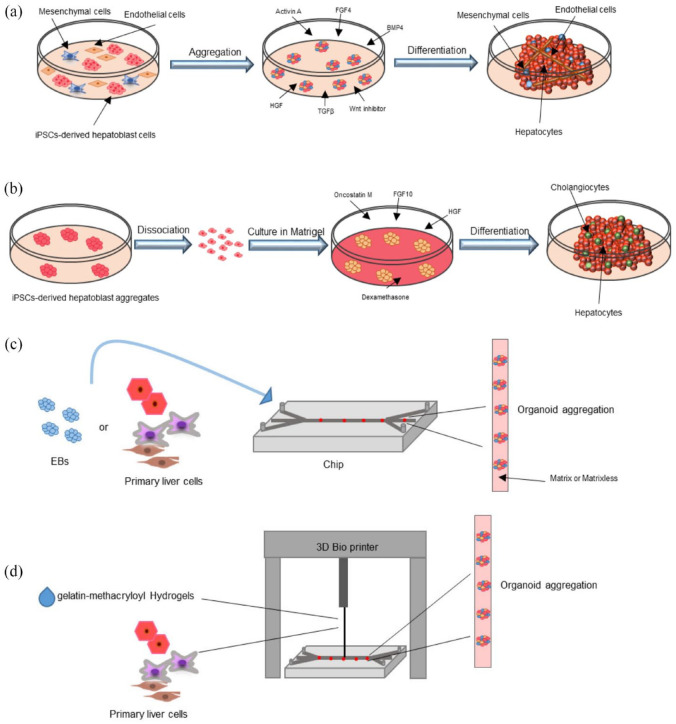
iPSCs-derived liver organoid generation. (a) utilizing hepatoblasts generated from iPSCs, mesenchymal cells, and endothelial cells in a co-culture system. After the initial aggregation, different cytokines are added to the culture media to aid in the creation of organoids. (b) developing organoids with only cells generated from iPSCs. Hepatoblast aggregates obtained from induced pluripotent stem cells (iPSCs) are distributed, cultivated in Matrigel with various cytokines, and differentiate into liver organoids that comprise cholangiocytes and hepatocytes. (c) using “Liver-on-a-chip” methods, which include growing primary liver cells or embryoid bodies generated from iPSCs on a chip in matrix-dependent or matrix-independent environments to encourage organoid aggregation. (d) Using primary hepatic cells and gelatin-methacryloyl hydrogels as ink, a three-dimensional (3D) printing technique is applied. These cells are then printed onto trans wells or perfused microwells to make liver organoids.^183^ (Reproduced from^183^ Cristina et al. 2020, Int. Journal of Molecular Sciences, under creative common attribution license CC-BY).

Organoids are in vitro biological systems that self-organize using mechanisms similar to those found in vivo. They replicate the shape and, in many cases, function of the in vivo tissue in question, thereby benefiting both therapeutic and fundamental research settings.^177^ Organoid models are increasingly being created using both adult primary cells and pluripotent stem cells (Figure 8).^152,178^

Further advances may enable organoids are envisioned to play a crucial role in regenerative medicine, with the ultimate aim of providing transplantable organs, seeding bio-artificial liver devices akin to kidney dialysis machines, or forming the active functional unit of an implanted device to offer temporary function. while relieving the burden on the liver’s highly regenerative capacity^153^ Current organoid systems are often carried out on a laboratory scale, which is incompatible with the previously stated desire for huge quantities of organoids. Some organizations have addressed the problem and begun to scale the method. However, because of the heavy reliance on recombinant growth factors, producing the required number of organoids is prohibitively costly. Further concerns in this area include the need to develop human-sized individual units of tissue, as illustrated by heart sheets, as well as the associated need for nutrients, and vascularization (either artificial or cell-based) that these larger models would require, given that current protocols generate organoids at the micrometer scale.^154^ Tissue engineering strategies may be thought of as employing organoids as essential building blocks for bigger organs, with ECM and/or biomaterials acting as scaffolds. Another rising concern is “Organ-on-Chip” technology, which combines organoid technology with chip-based technology’s control and automation features. Furthermore, a platform for characterizing and analyzing organoids function in an “online” manner is supplied, allowing multiple tissue types (organoids) to be grown in the same systems, assisting in the development of more complicated models.^179^

### Adult liver organoids

Epithelial cell adhesion molecules (EPCAM+), biliary epithelial cells were used to create liver organoids from primary cells, building on recent work with a Stem cell population expressing Leucine-rich-repeat containing G-protein coupled receptor-5 (LGR5+), isolated from the gut.^180^ They demonstrate that these organoids, arranged as polarized cysts, replicate some of the epithelial properties of the original organ, and that, despite being produced from ductal cells, the organoids have hepatobiliary traits and can be guided to a hepatocyte-like phenotype.^181^ These organoids were clonally generated, grew, and remained viable for several months. More recent studies in the field have created hepatocyte-derived organoids (Hep-Orgs) from both adult and fetal liver, resulting in a more defined hepatocyte (rather than biliary or hepatobiliary) phenotype. These include multidrug resistance associated protein-2 (MRP2) positive bile canaliculi formation, tight junction integrity, albumin secretion, and CYP3A4 activity levels similar to those of primary human hepatocytes (PHHs).^158^ Alpha feto protein (AFP) secretion levels were initially high but decreased with time, supporting the author’s idea that the Hep-Orgs develop in a way similar to the liver’s regeneration response, as fetal AFP secretion is likewise increased following hepatectomy. A lack of multidrug resistance protein-1 (MDR1) activity in the liver tissues indicated the absence of cholangiocyte function, whereas only a small number of cholangiocytes were generated.^156^

The lack of diversity in early populations is a constraint of adult cell-generated organoids; because they are derived from epithelial cells, there is no potential for endothelial or mesenchymal cell types, which might aid in developing complexity and a more organotypic model. This has implications for their prospective use in modeling disorders including fibrosis, MASLD, and HCC, all of which include inflammation and the cellular milieu, as well as growth, which necessitates the presence of several cell types and lineages. Their capacity to reproduce monogenic illnesses such as allagile syndrome (ALGS) and Alpha-1 antitrypsin (A1AT) deficiency has previously been proven.^157,181^ Since they are produced from epithelial cells, they eliminate the possibility of endothelial or mesenchymal cell types, perhaps leading to increased complexity and an organotypic model. More research is needed to determine whether the primary-derived organoids develop through a process that more closely resembles regeneration rather than mere improvement. Hep-Orgs aid in evaluating this aspect and determining if a more tissue-like parenchymal model, incorporating both hepatocytes and cholangiocytes organized similarly to in vivo conditions (such as with duct structures), can be created.^157^

**Figure 9.**
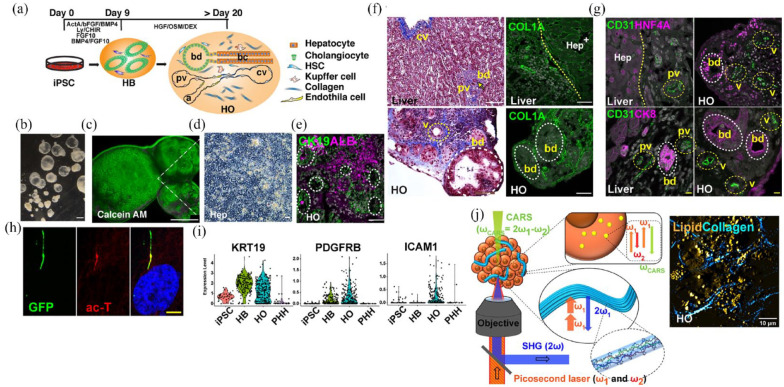
Human multi-lineage Hepatic Organoid model forms complex structures that resemble those in human liver. (Reproduced with permission from^182^ Guan et al. 2021, Nat Commun,^12^) (a) A schematic representation of the in vitro culture system that directs IPSC to differentiate into HOs. The following structures are indicated in the images: cv, central vein; pv, portal vein; and bd, bile duct; bc, bile canaliculus; a, artery. (b) A low-power, bright field view of HOs obtained after 21 days of differentiation. Scale bar is 500 μm. (c) Calcein AM staining indicates that cells within an organoid are viable, the Scale bar is 500 μm. (d) A high-power bright field image of the region indicated in (c) shows the polygonal hepatocyte morphology of the cells within an HO. These cells also have lipid vesicles, which appear as bright areas. (e) Immunostaining shows Albumin+ hepatocytes and CK19+ cholangiocytes within the HOs. The dotted circles indicate bile ducts. Scale bar is 50 μm. (f) Left: Trichrome staining shows that some of the structures present in normal liver (top) are also present in HOs (bottom). Right: Immunostaining shows that collagen is present in peri-ductal and vascular areas. The yellow dotted line delineates an area with hepatocytes (Hep+) in normal liver. Scale bars are 50 μm. (g) HOs were immunostained with antibodies to endothelial cell (CD31), and hepato-biliary (HNF4A, CK8) markers. Structures resembling bile ducts (bd), portal vein (pv), and venules (v) are present in HOs. Scale bars are 50 μm. (h) A primary cilium in a day 21 HO was visualized with an ARL13B-GFP fusion protein (GFP), and by immunostaining with acetylated tubulin (ac-T). Scale bar is 5 μm. (i) scRNA-seq data indicates that HOs express multi-lineage markers, which include CK19 (Cholangiocyte), PDGFRB (hepatic stellate cells), and ICAM1 (endothelial cells). (j) Left: A schematic diagram of SHG and CARS microscopic imaging of a HO with collagen fibers (cyan).

Specific growth factor combinations added successively to the culture media cause iPSCs to differentiate into hepatoblasts, which subsequently become hepatic organoids (HOs) (Figure 9(a)–(d)). Hepatocyte sheets and arranged cholangiocytes produce epithelia around lumina resembling bile ducts in these HOs (Figure 9(e)). Functional tests have demonstrated that these HOs can carry out a wide range of drug metabolism and biosynthesis processes typical of the human liver (Figure 9(f)). The mesenchymal tissue within HOs closely mimics native liver tissue, as shown by Trichrome staining and collagen (COL1A) immunostaining in liver slices (Figure 9(f)). Remarkably, the pictures imply that HOs express a more sophisticated antigen pattern and create more complex structures than was previously known. The organoids show collagen-rich ECM, and ductal structures encircled by mesenchymal cells; CD31 staining indicates the existence of vascular structures within HOs (Figure 9(g)). Furthermore, HOs’ bile ducts exhibit primary cilia, which are crucial for examining ciliopathic illnesses (Figure 9(h)). Hepatocytes, cholangiocytes, and bipotential progenitor cells that can differentiate into both hepatocytes and cholangiocytes are among the distinct cell clusters that can be found using the single-cell RNA sequencing (scRNA-Seq) technique^182^

### Hepatobiliary organoids

A more physiological model is proposed, in which hepatobiliary organoids are created by modifying existing hepatocyte differentiation to encourage the development of both endoderm and mesoderm tissues within the same culture. Transforming Growth Factor (TGF) stimulation drives the differentiation of hepatocyte and cholangiocyte populations from a specific source. These hepatobiliary organoids are cultivated in a standard base medium enhanced with a proprietary cholesterol supplement (MIX) 160. This model captures certain stages of hepatogenesis, including the formation of bipotent precursors and their subsequent differentiation into both lineages (along with the formation of endothelial cells), as well as the organization of these cell types into structures like lumens surrounding cysts and tubes has apical-basal polarity, cilia, and bile acid in cystic forms. This demonstrates a functional multicellular system, since hepatocytes produce bile before it is released and pooled in the ductal cavity. Biliary cells formed rings and then converted into tubular or cystic structures when they expanded out of the culture. The development of these structures was revealed to be associated with NOTCH signaling. It was thought to be related with the previously stated endothelial cells, comparable to the crosstalk between cell types observed during development in vivo, where the mesenchyme performs this job.^157^ The resultant culture has been revealed. The resultant culture included higher levels of maturation markers for both the hepatocyte and biliary lineages. Nonetheless, they had the same limitations as many iPSC-derived children, with low levels of CYP450 activity that matched or exceeded fetal liver levels but were only a quarter of adult liver levels.^156^ The above model corresponds to the organoid description, including advanced multi-cell features such as bile production, flow, and concentration in tissue approximation structures. Unlike more conventional ways of embedding cells inside an ECM, the cells were cultivated as a monolayer and allowed to overgrow throughout maturation, resulting in separate 3D masses. This approach stresses the lack of supporting mesenchyme or fibroblast cell types, as well as the ECM or biomaterial, resulting in decreased cost and complexity. However, the lack of mesenchymal cell types may hinder the formation of a complete organoid.^184^

The advancement of liver organoid models addresses the limitations of traditional 2D cell cultures by offering a more physiologically relevant representation of liver tissue. Current liver organoid models often rely on expensive growth factors, ECMs, and 2D patterning, resulting in simplified and less representative structures. A new approach has been developed that bypasses 2D patterning and ECM dependency, using small molecules to mimic liver development. This method produces large quantities of liver-like organoids with complex cellular structures, including hepatocytes, cholangiocytes, Kupffer cells, and vascular components. These organoids exhibit key liver functions such as drug metabolism, protein production, urea synthesis, and coagulation factor production. They can be transplanted into mice, where they produce human albumin and show signs of de novo vascularization. The new protocol is efficient and scalable, producing up to 500 organoids per milliliter in a cost-effective manner. This advancement holds promise for applications in cellular therapy, tissue engineering, drug testing, disease modeling, and basic biology. Future work will focus on further scaling, improving standardization, and exploring the organoids’ potential in transplantation and disease research.

### Applications of liver organoids

Organoids have evolved as relevant variables for biomedical research. Liver organoids have a variety of uses in fundamental research and therapeutics, including liver regenerative medicine, disease modeling, drug screening, and individualized therapy. Several liver organoids have been produced for a variety of uses.

#### Liver regeneration

A study found that organoids derived from mouse Lgr5 liver stem cells could be implanted into Fah-/- mutant mice (models of tyrosinemia type I liver disease) to evaluate their therapeutic potential. The Fah-/- mutant mice did not survive without the administration of NTBC (2-(2-nitro-4-trifluoromethylbenzoyl)-1,3–cyclohexanedione). Liver organoids matured into hepatocytes in vitro. Although mice survival increased 2 months after transplantation, Fah nodules only accounted for 1% of liver mass. Low engraftment capability has been associated with lower therapeutic efficacy.^158^ Furthermore, human EPCAM ductal cell-derived organoids were implanted into CCl4-treated nude mice, resulting in significant liver injury. Seven days after transplantation, human albumin (hALB) and alpha-1-antitrypsin were detected in mouse serum. For 120 days, the levels of hALB and alpha-1-antitrypsin remained stable. These levels were estimated 1 month after human PH transplantation. Peng et al. recently revealed that mouse PH-derived organoids had strong engraftment potential. In Fah-/- mutant mice, the number of Fah cells increased by up to 80% 103 days after implantation.^157^ Another study injected human pH-derived organoids into immune-deficient Fah-/- NOD Rag1-/- Il2rg-/- (FNRG) mice. The organoids grafted at a rate comparable to PHs 30 days after transplantation. The hALB of serum was around 200 mg/ml 90 days after donation.^184^

#### Liver disease models

Diseases provide a foundation for the creation of successful treatments. A1AT deficit is a hereditary metabolic disease characterized by A1AT deficiency in serum. Alagille syndrome is marked by the absence of interlobular bile ducts, which results in severe cholestasis and cirrhosis.^185^ A group of researchers the researchers were successful in creating liver organoids from liver biopsies of people with A1AT deficiency and Alagille syndrome. These organoids recreated in vivo pathology. Organoids derived from A1AT-deficient patients showed increased ALB levels and low-density lipoprotein intake. Organoids from patients with Alagille syndrome, on the other hand, showed aberrant biliary tree anatomy. Wilson’s disease is an extremely uncommon inherited copper storage disorder.^186^ A recent study found that canine liver organoids from Copper Metabolism MURR1 Domain-containing Protein 1 (COMMD1) -deficient puppies accumulated more intracellular copper, which was very similar to Wilson’s disease in vivo^187^Citrullinemia type 1 (CTLN1) is a urea cycle disorder marked by abnormal arginosuccinate synthetase (ASS) enzyme activity. Akbari et al. created liver organoids using patient-specific iPSCs. Ammonia buildup in organoids was linked to CTLN1 dysfunction. Furthermore, organoids restored the CTLN1 phenotype via ectopic production of the ASS1 enzyme.^188^

#### Drug screening and personalized treatment

Only a few models accurately mimic the pathophysiology of actual tumors in vitro, restricting the development and delivery of therapeutics.^189^ According to the FDA, more than 90% of safe medications that performed well in animal testing failed in human clinical trials. Drug research is currently characterized by tremendous input but low output, suggesting that only a few medications make it through clinical trials.^190^ The introduction of organoid technology represents a promising avenue for medical advancement. It is increasingly being used for preclinical drug screening and forecasting individual patient treatment results. Liver organoids produced from iPSCs, and ASCs can be used to represent a variety of illnesses in vivo. As a result, organoids could be used in future drug screening and precision medicine. Furthermore, studies are being conducted to create cryobanks of healthy and sick human organoids for biomedical purposes, which can replace typical 2D cell lines with patient derived xenograft model (PDX) for drug screening.^186^ A team of researchers successfully developed 27 primary liver cancer (PLC) organoid lines. These organoids produced 3483 data points regarding cell survival. According to this study, most medications were useless; nevertheless, a small percentage had a minimal influence.^191^ Using PLC to manufacture liver tumor organoids enables more personalized treatment on a smaller scale. Another study found that PLC-derived organoids could be used to evaluate potential novel target treatments and as laboratory imitations for medication evaluation in personalized medicine.^192,193^

An in vitro model suitable for predicting DILI risk uses hepatic organoids (HOs), which are compatible with high-throughput screening methods and can be modified into Precision-cut Liver Organoid Cultures (PaDLOCs) to improve their organotypic functionality Compared to primary human hepatocytes (PHHs), HOs present advantages in scalability and consistency, facilitating large-scale and high-throughput DILI risk assessment, particularly when employed in a 384-well format for early-stage preclinical evaluation of novel drugs. PaDLOCs exhibit physiological similarities to the human liver, including the production of hepatocyte-like and stellate-like cells from the same host genetics, albumin production, and expression of CYP450 enzymes. HOs, when cultured as a dispersed monolayer, reduce variability between wells and enable clear single-cell resolution imaging, facilitating high-content screening, Cell painting, and morphological cell profiling. This approach allows for the clustering of drugs based on their phenotypic effects, enabling comparisons and inference of similar mechanisms of action through multivariate analysis. Additionally, machine learning-based algorithms can integrate multiple features to generate robust prediction scores.^167^

Despite the advantages of high-throughput screening with dispersed HOs, they exhibit lower CYP450 expression and lack certain crucial hepatocyte functions compared to PHHs. However, PaDLOCs, which comprise a mixture of parenchymal and non-parenchymal cell types, respond to drugs at in vivo concentrations, likely due to more accurate drug metabolism. In studies, hepatotoxic effects of drugs such as FIAU and tenofovir-inarigivir were observed across multiple cell lines at clinically relevant concentrations, which were not detected until clinical trials or at higher concentrations in the 384-well platform. Moreover, histological observations of drug treated PaDLOCs closely resembled pathological features observed in patient liver tissues.^194^

Integration of scRNA-seq with liver chip systems enhances predictive capabilities for synergistic DILI. For example, a unified multi-omics platform supported by transcriptomics data predicted the synergy between FIAU and tenofovir-inarigivir, which was confirmed in dose-response assays. PaDLOCs, being iPSC-based, offer the potential for expanded modeling encompassing patient genetic diversity, paving the way for benchmarking compounds before clinical trials and mitigating rare hepatotoxic events. Future studies aim to develop a biobank of complex HO co-cultures derived from well-phenotyped and genotyped cases of idiosyncratic DILI, focusing on enhancing DILI risk assessment by concentrating on genetics within a screen able number of patient lines.^182,194,195^

**Figure 10.**
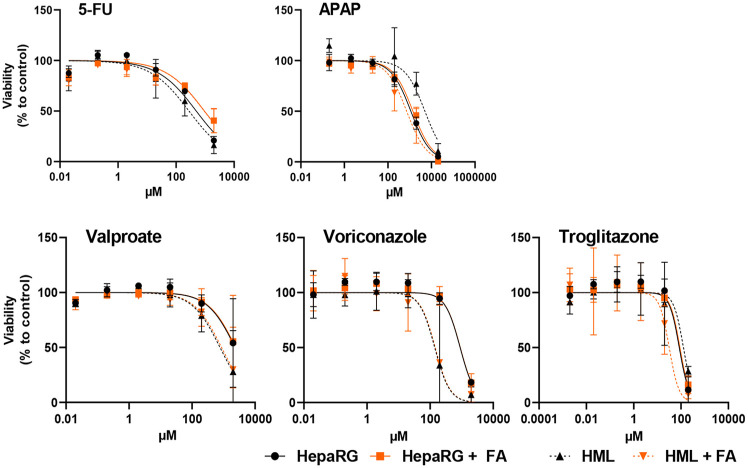
Evaluation of Drug toxicity. analyzing the toxicity of the medication after nine days of exposure on steatotic HepaRG cells and HML organoids. On day 5, HepaRG cells and HML organoids were exposed to a fatty acid combination. The medium was then supplemented with 5-fluorouracil (5-FU), paracetamol, valproate, voriconazole, or troglitazone from Day 7 to Day 14. Intracellular ATP levels were measured to evaluate the levels of toxicity.^195^ (Reproduced from^195^ Bronsard et al. 2024, Toxicology Invitro under creative common attribution license CC-BY-NC).

In this study, liver functions have been successfully maintained in vitro through 2D co-cultures, demonstrating activity in xenobiotic-metabolizing enzymes. Notably, HepaRG progenitor cells exhibit differentiation into both cholangiocyte-like and hepatocyte-like cells, each displaying liver-specific functions. In efforts to replicate immune-mediated hepatotoxicity and investigate inflammatory stress implications, co-cultures of macrophages and HepaRG have been employed. Additionally, 3D co-cultures involving HepaRG cells with primary HSCs have been utilized to explore liver fibrosis. Here, a novel HML 3D liver organoid model has been developed, encompassing differentiated HepaRG cells, HSC-derived LX-2 cells, and macrophages. These organoids successfully simulate a MASLD-like state and demonstrate sensitivity to drug-induced hepatotoxicity. Through chronic exposure studies, it was observed that the HML organoids maintain stable liver marker expression levels, enabling prolonged investigations. Importantly, the model shows promise in reflecting the exacerbated cytotoxicity of drugs like paracetamol and troglitazone in MASLD conditions. Conversely, drugs like 5-FU, voriconazole, and valproate exhibit consistent cytotoxicity across MASLD and non-MASLD conditions. These findings underscore the potential utility of HepaRG cell based MASLD models in screening drug hepatotoxicity, aiding in future clinical investigations (Figure 10).^195^

## Conclusion

Finally, this review investigates several therapeutic techniques within the realm of hepatic tissue engineering. The utilization of polymer-based scaffolds, which includes both natural (e.g. Chitosan, Gelatin, dECM, Alginate) and synthetic polymers (e.g. PCL, PVA, PEG, PLGA), highlights a multidimensional approach to scaffold development. The range of fabrication techniques, from traditional methods to advanced 3D bioprinting, reflects the continuous evolution of tissue engineering methodologies. The incorporation of diverse cell types into bioinks for 3D bioprinting underscores the complexity of replicating native liver architecture. Additionally, innovative therapies such as Liver-On-A-Chip, liver organoids, and spheroids expand the spectrum of treatment possibilities. Collectively, these advancements mark a promising trajectory for liver tissue engineering, offering varied therapeutic options and advancing our understanding of liver biology and pathology.

However, despite these promising developments, there are several limitations to the current findings. Many scaffold and bioprinting techniques are still in the experimental phase, with challenges related to scalability, reproducibility, and integration with existing medical practices. The functional maturity of engineered liver tissues often falls short of fully replicating the complex functions of native liver tissues, including long-term stability and comprehensive metabolic activities. Additionally, the use of synthetic materials and advanced bioinks can sometimes lead to issues with biocompatibility and inflammation. There is also a need for more extensive in vivo studies to assess the long-term performance and safety of these engineered tissues. Addressing these limitations will be crucial for translating these innovations into practical, clinical applications and achieving significant improvements in liver disease treatment and organ replacement.
